# Streaming visualisation of quantitative mass spectrometry data based on a novel raw signal decomposition method

**DOI:** 10.1002/pmic.201400428

**Published:** 2015-03-09

**Authors:** Yan Zhang, Ranjeet Bhamber, Isabel Riba-Garcia, Hanqing Liao, Richard D Unwin, Andrew W Dowsey

**Affiliations:** 1 Centre for Endocrinology and Diabetes, Institute of Human Development, Faculty of Medical and Human Sciences, The University of ManchesterManchester, UK; 2 Centre for Advanced Discovery and Experimental Therapeutics (CADET), Central Manchester University Hospitals NHS Foundation Trust, Manchester Academic Health Sciences CentreManchester, UK

**Keywords:** Bioinformatics, Mass spectrometry, Quality control, Signal decomposition, Visualization

## Abstract

As data rates rise, there is a danger that informatics for high-throughput LC-MS becomes more opaque and inaccessible to practitioners. It is therefore critical that efficient visualisation tools are available to facilitate quality control, verification, validation, interpretation, and sharing of raw MS data and the results of MS analyses. Currently, MS data is stored as contiguous spectra. Recall of individual spectra is quick but panoramas, zooming and panning across whole datasets necessitates processing/memory overheads impractical for interactive use. Moreover, visualisation is challenging if significant quantification data is missing due to data-dependent acquisition of MS/MS spectra. In order to tackle these issues, we leverage our seaMass technique for novel signal decomposition. LC-MS data is modelled as a 2D surface through selection of a sparse set of weighted B-spline basis functions from an over-complete dictionary. By ordering and spatially partitioning the weights with an R-tree data model, efficient streaming visualisations are achieved. In this paper, we describe the core MS1 visualisation engine and overlay of MS/MS annotations. This enables the mass spectrometrist to quickly inspect whole runs for ionisation/chromatographic issues, MS/MS precursors for coverage problems, or putative biomarkers for interferences, for example. The open-source software is available from http://seamass.net/viz/.

## 1 Introduction

MS/MS has become the pervasive technique for access to the proteome and metabolome. The demand for faster and deeper analysis has resulted in progressive technology advancements that have escalated the rate and size of data output tremendously, and this is unlikely to slow [1]. Moreover, MS practitioners continue to plan larger and more multifaceted experimental designs to attain power and sensitivity to confidently answer complex biological questions in systems biology and medicine. While the bioinformatics and biostatistics fields are endeavouring to keep pace with this data explosion with more intricate and automated analyses to interpret the data, there is a danger that the process becomes more and more opaque and inaccessible to MS practitioners and so more likely to be used as a ‘black box’. It is therefore vitally important that tools and platforms are available that allow expert user verification, validation and interpretation of results in the context of the raw acquired spectra, otherwise bias, errors and false assumptions in acquisition and processing will be routinely overlooked.

Visualisation techniques constitute a core aspect of biological data analysis, for they provide a direct and user-friendly means to gain insight and interpretation of the properties of the data. Starting with the inspection of the raw data, visualisation methods assist in exploring the experimental results more effectively than by simply examining numbers in large tables and lists [2,3], which lack the spatial organisation and conceal the quality control aspects that the human eye can easily recognise. It is widely acknowledged that integrated data and results visualisation is of significant benefit to interpretation in proteomics and metabolomics [1,4,5] supporting quality control for the identification of issues and artefacts caused by experimental design, acquisition and processing. Here, human cognition is harnessed to assist users in understanding the underlying phenomena and casual relationships in their data. Most commercial and academic LC-MS software packages therefore contain a variety of 1D, 2D and 3D visualisations of varying sophistication.

LC-MS data have three or four components: chromatographic retention time (RT), *m/z*, ion count (intensity) and more recently, ion mobility. Manual data interpretation is currently predominantly performed using 1D visualisation with mass spectral plots (intensity over *m/z* for a specific RT) and extracted ion chromatograms (XICs: intensity over RT for a specific *m/z*). Annotated 1D spectral plots are the mainstay of visual interpretation, particularly for MS/MS fragmentation spectrum appraisal, e.g., for identifying PTMs, or de novo sequencing [6]. Nevertheless, exploring the data by looking at 1D plots does not lead to an optimum comprehension of the quality of quantitative data. Instead, one would like to have a complete view across *m/z* and RT, i.e., examining intensities across a 2D domain. Image-based visualisation and interpretation is the natural method to handle 2D electrophoresis gels. For LC-MS data, the first 2D ‘virtual gel’ representation was proposed by Li et al. [7]. These days, typical visualisations aim to integrate raw data with peak segmentation and quantification results, and with MS/MS product ion spectra and identification results through annotation of their precursors [8]. They can also incorporate differential displays or tilings to compare fractions or biological replicates. For both LC-MS and GC-MS, 3D topographical ‘terrain rendering’ has been demonstrated as a valuable addition [9]. More advanced cognitive visualisations have also recently be proposed, for example with annotated pathway analysis results [10] and where metrics are encoded by shape, size and colour of glyphs [11]. Finally, it is perhaps not surprising that MS Imaging data in particular has spawned a wealth of visualisation research, where dimensionality reduction, segmentation and false colour have been used to create ‘virtual histopathology’ maps to aid clinical diagnosis [4].

Since the Proteomics Standards Initiative mzML data interchange format [12] organises full-scan data as a contiguous list of raw spectra, recall of individual spectra is fast due to the indexing scheme and their relatively small size. However, a common task is to view XICs to assess the chromatographic separation of a biochemical. For datasets not stored as XICs (i.e., non-SRM data), visualisation requires every MS spectrum in the dataset to be extracted. Moreover, generating a 2D image (‘virtual gel’) of an LC-MS dataset requires every single datapoint to be loaded, despite the limited pixel density of the user's display. To mitigate some of these issues, 3D visualisations in the commercial Progenesis package (Nonlinear Dynamics, Waters Inc.) are rendered from small regions of interest around delineated peaks. However, the rendering is still not instantaneous, thus restricting productivity and user motivation.

Current streaming visualisation technologies for large-scale spatial data such as Deep Zoom (http://www.microsoft.com/silverlight/deep-zoom/), Zoomify (http://zoomify.com/) and Google Maps (http://maps.google.com) use the image pyramid as a basic building block for displaying large images in an efficient way. A typical image pyramid decomposes an image at multiple dyadic resolutions (i.e., multiscale), and at each resolution the image is tessellated into axis-aligned tiles. For visualisation, the resolution closest to the viewport resolution is selected and only visible tiles are selected for display. Setting the parameter values of the pyramid such as the number of levels and tile size allows control of the data transfer rate. Attaining reasonable streaming performance requires each tile to be compressed with a progressive coding scheme so that a coarse version of each tile is displayed as soon as possible and then iteratively refined as more data is received.

There are several issues with these approaches for visualising LC-MS data. Firstly, MS analysis should not be compromised by lossy image compression methods that make assumptions based on the acuity of human vision. Secondly, progressive image compression ranks image features for display by their spatial extent, but in MS peaks are more cognitively important than background regions, yet have very localised extent. Thirdly, compression of each separate tile leads to visible discontinuities at tile boundaries until the data is fully loaded. Finally, MS datasets are not structured over a regular Cartesian grid like conventional images or computer monitors: irregular sampling in the *m/z* dimension depends on the Orbitrap signal intensity or the TOF quadratic calibration equation [14], whereas data point position in the RT dimension depend on any adaptive scan times of prior MS/MS acquisitions. Tools that generate ‘virtual gel’ images need to ensure that no inaccuracies are produced by the resampling or rebinning process, particularly when displaying peak apexes at accurate mass.

While issues surrounding dataset loading have not been tackled so far, streaming and display of data already residing in memory has been studied. 3D approaches are based on multiresolution terrain modelling with on-demand view-dependent rendering [15]. A tree or graph-based mesh is designed so that inhomogeneous resolutions can be extracted at run-time based on a curvature and/or image-based error criterion. Lossaso and Hoppe's seminal approach [16] presents an optimised GPU representation where the meshes are fixed based on the level-of-detail (LOD) reducing in increasingly sized rectangular hoops centred on the viewer. However, cracks requiring stitching appear between LODs. Since displayed LOD is purely view dependent and not based on cognitive importance of peaks, Corral and Pfister [17] decided to compute the maximum rather than average intensities at each LOD. This mitigates the problem for peaks in the foreground, but as a side effect peaks widen as they become more distant. In more recent work for metabolomics, Linsen et al. [14] use a maximum intensity wavelet decomposition equivalently, but only display a single LOD.

In this paper, we describe a next-generation engine for fast and flexible visualisation of large-scale raw quantitative LC-MS data. By decomposing the MS signal into its constituent components and ordering these parts by their cognitive importance, we are able to provide accurate visualisations at low data rates: Early approximate reconstructions permit fast response to initiation, panning and zooming, while later reconstructions reveal detailed peak shape and tertiary structures. The intention is that our platform can replace the simplified raw data handling currently adopted by current viewers, fusing our fast streaming visualisations with their existing advanced annotation overlays.

## 2 Materials and methods

**Figure 1:**
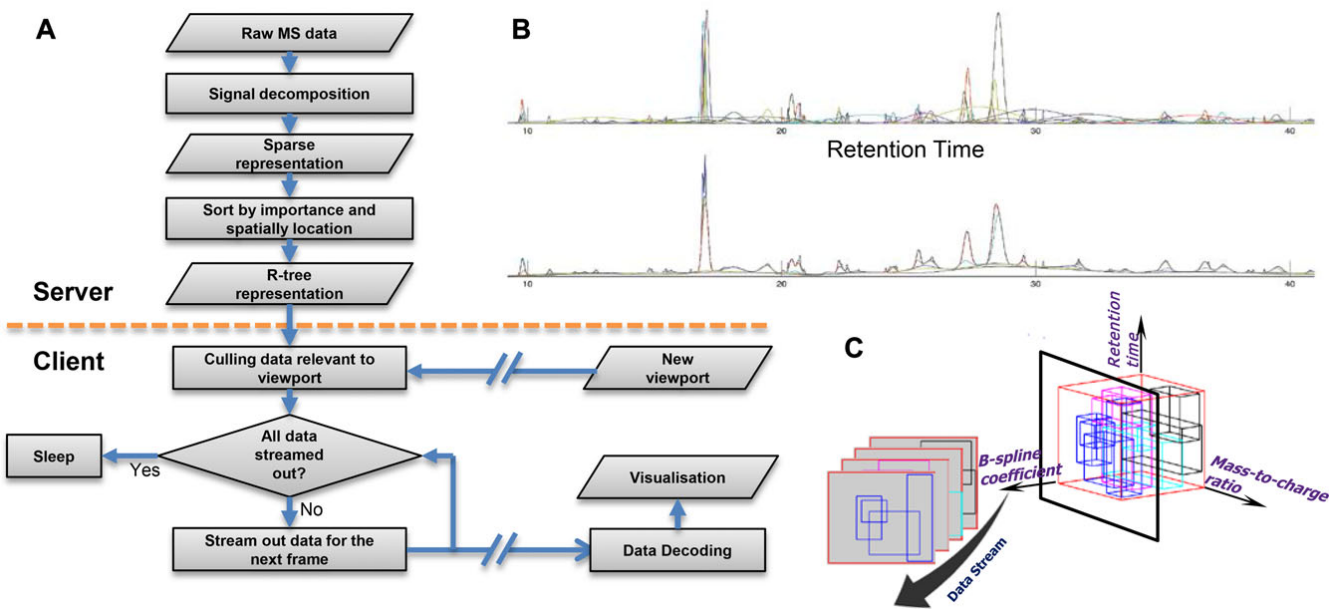
(A) Flow chart of the proposed asymmetric visualisation platform for LC-MS data. A server or workstation is employed to decompose the dataset and store the result in an R-tree format for archival. Lightweight clients can then efficiently read and visualise this format interactively. (B) Top: A sparse set of multiscale B-spline basis functions selected by seaMass to accurately fit an XIC. Bottom: The client simply adds these basis functions up to reconstruct the XIC for visualisation. (C) The basis functions are partitioned by *m/z*, RT, and weight coefficient using an R-tree index structure. These are streamed out to the client's display in decreasing order of weight.

As illustrated in Fig. 1A, we propose an asymmetric scheme for providing fast visualisation of raw profile-mode LC-MS data. Initially, each dataset is pre-processed with our signal decomposition algorithm into a sparse set of image ‘building blocks’, which are then indexed both spatially and by their cognitive importance through an R-tree representation and stored back to disk. This is a computationally intensive process but is only required once and therefore has been implemented as a ProteoWizard [18] filter so that it can be performed in parallel with conversion to mzML, e.g., on acquisition or on upload to a public repository. Subsequently, our lightweight viewer can interactively visualise this data rapidly and efficiently. To facilitate interpretation of the data, the viewer harnesses the ability of the signal decomposition algorithm to adaptively interpolate missing MS1 spectra. MS1 spectra are missed when the instrument is switched into isolation mode to acquire MS/MS or MS^n^ identification spectra. This is particularly noticeable with data-dependent acquisition (DDA), where the instrument selects the most intense MS1 peaks for isolation, fragmentation and analysis.

### 2.1 Sparse signal decomposition for LC-MS data

Sparse signal decomposition approaches rely on the ability to deconstruct a signal parsimoniously into a weighted linear combination of elementary signals, or atoms, from a dictionary of such signals [19]. In the LC-MS informatics field, these methods have so far been utilised extensively for denoising [20] and feature detection [21]. For denoising, multiscale wavelet dictionaries are extremely popular due to their mathematical properties that enable an optimal decomposition to be calculated in linear time under a Gaussian noise assumption. Wavelet denoising works on the principle that smooth structured signal can be represented by a sparse (small) set of shifted and scaled wavelets of large weight, while Gaussian noise is distributed weakly across wavelets of all shifts and scales. Most noise can therefore be removed by thresholding minor weights to zero.

Since the greater the weight, the greater the contribution to the overall signal, the same technique is valuable for signal compression and prioritising coefficients for streaming visualisation. However, we believe that Gaussian wavelet methods are not appropriate for streaming visualisation of LC-MS data. Firstly, a recent study on TOF instrumentation has shown that ion counting statistics dwarf other noise components [22]. This suggests a Poisson noise model rather than Gaussian, which immediately offers two advantages: Firstly, negative counts are unattainable. Secondly, Poisson variance is equal to its mean, which is supported by observations that MS signal variance drops as *m/z* increases in linear TOF instruments, since the mean data-point intensity decreases due to peak spread [23]. Note that while Orbitrap instruments sample ion counts indirectly through induction of a current that captures the frequencies of oscillation of all ions simultaneously, the statistics of discrete and finite ion counts still ensure that the signal has an underlying pseudo-Poisson distribution.

Secondly, wavelet filters have zero mean by design. This enables wavelets at each scale to approximate the signal without introducing bias: Wavelets of finer scale improve on the signal approximation by adding to and subtracting from it in equal measure. Therefore, LC-MS visualisation with a wavelet decomposition would lead to early approximate reconstructions exhibiting erroneous ion signals that would then be removed by later refinements. Conversely, each atom in our multiscale B-spline basis function dictionary is restricted to have a non-negative weight by the Poisson model. In this way, they act as ‘building blocks’ that additively construct the LC-MS signal. We believe that multiscale uniform B-spline basis functions of 3^rd^ order (cubic) have the ability to model generic LC-MS signals closely: As shown for an XIC decomposition in Fig. 1B, uniform B-spline basis functions approximate Gaussian peaks, but unlike Gaussians are not infinite in width (in fact, B-spline basis functions converge to Gaussian functions as the order increases to infinity). Furthermore, background signal and peak shoulders can be modelled by consecutive B-spline basis functions spaced at 1/(order+1) intervals, which is mathematically equivalent to the piecewise polynomial of global continuity with shortest support, which is called a B-spline curve [24].

In our *seaMass* software, we adopt an overcomplete set of separable tensor-product B-spline basis functions to efficiently model correlated signals across *m/z* and RT dimensions. We treat the raw input data as irregularly spaced ion count bins. Discrete kernels are generated to map the continuous B-spline basis functions onto these discrete bins. In effect, this fits a continuous, maximally smooth 2D surface to the raw data so that missing regions (due to DDA MS/MS acquisition) are implicitly imputed. The algorithm relies on shrinkage hyper-parameter *λ*, which controls the trade-off between fitting accuracy and the size of the resulting sparse coefficient set. For further technical details of the methodology, please see [25].

### 2.2 R-tree representation for data streaming

To retrieve the B-spline coefficients spatially and by cognitive importance within a tolerable latency time, an efficient spatial data index structure is necessary. As two popular methods, Octree [26] is widely used in graphics applications, while the R-tree [27] is the most commonly employed spatial indexing structure in the database community. Both can represent 3D data and therefore organise the B-spline basis functions from the sparse decomposition both spatially in *m/z* and RT, and by basis function weight, which is our proxy for cognitive importance.

An Octree is a 3D data generalisation of a binary tree. Each node represents the volume formed by an axis-aligned 3D cuboid and has up to eight children, each corresponding to one equally sized octant of the parent cuboid. While Octrees provide extremely fast spatial querying, their encoding is suboptimal when the encoded data is not spread evenly across the three dimensions. Moreover, since Octrees can index only regions of fixed size, we would need to construct a different Octree to encode the coefficients of each scale of B-spline basis function.

The R-tree is an extension of the one-dimensional B-tree for multi-dimensional data. As illustrated in Fig. 1C, 3D R-trees provide a spatial index of axis-aligned cuboids of any size by deriving hierarchically nested and possibly overlapping containing cuboids. The tree is height balanced, with each node corresponding to one hard disk chunk (e.g., 4kb) for optimal random access. Several libraries exists that implement R-tree for storing and processing spatial data outside of databases. For performance considerations we adopted the lightweight libspatialindex library (http://libspatialindex.github.io/), which provides improved R* splitting heuristics for improved query efficiency [27].

### 2.3 Lightweight visualisation client with dynamic multiresolution coring

Data flow in the visualisation client is illustrated in Fig. 1A. For each desired viewpoint, the R-tree index is queried and the spatial locations of a packet of basis functions are delivered in order of their weight. Delivery of multiple consecutive packets enables dynamic multiresolution coring during the decompression process: Main image features, i.e., intense peaks and background, will be displayed in initial frames, with finer details built up incrementally. To minimise computation, coarser B-spline basis functions are transformed to a single-scale B-spline surface at the scale closest to the viewport resolution using a convenient property that enable them to be transformed between scales analytically [34]. This surface is then sampled to the display, before the remaining finer scale basis functions are rasterised directly.

## 3 Results

We demonstrate the usage of our visualisation platform on exemplar ToF and Orbitrap datasets. The Clinical Proteomic Tumor Analysis Consortium (NCI/NIH) generated Orbitrap data used in this publication. LC-TOF-MS data from an Agilent 6530 Q-TOF was acquired in CADET, University of Manchester. The datasets are available for download at http://seamass.net/viz/.

**Figure 2:**
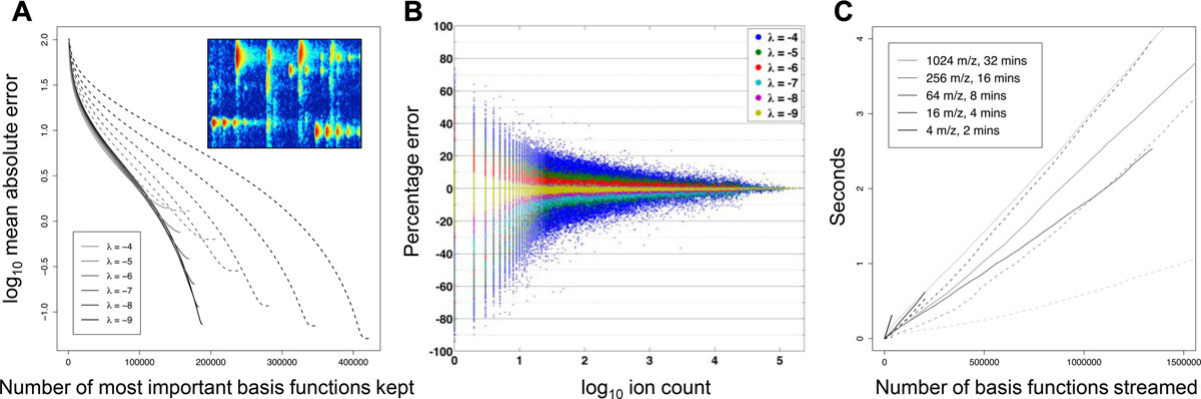
(A) For the small TOF region illustrated, comparison of the mean absolute error between the raw data points and the incremental addition of basis functions to the visualisation by decreasing order of importance. Curves show seaMass sparse decompositions with shrinkage hyperparameter *λ* between 2^−4^ and 2^−9^. Dotted and solid curves represent results after 100 and 2000 seaMass iterations, respectively. (B) For the completed visualisations with *λ* between 2^−4^ and 2^−9^, the percentage error between seaMass and the raw data point is plotted against the magnitude of each raw ion count. (C) For a complete TOF dataset, streaming rates for R-tree reading from disk (solid curves) and image reconstruction for display at 800 × 600 (dashed curves) for viewport sizes from overview to deep zoom.

Initially, we objectively assessed how close the reconstructed incremental streaming visualisations are to the original raw data. This is dependent on the shrinkage hyper-parameter *λ* used in the sparse signal decomposition algorithm. As shown in Fig. 2A, if the iterative algorithm is run exhaustively (2000 iterations, solid curves), a wide range of the shrinkage parameter (2^−4^ to 2^−9^) leads to similar mean absolute errors between the raw and reconstructed data points as more basis functions are utilised, except that lower shrinkage values lead to a final reconstruction with less error. However, running seaMass on complete datasets for 2000 iterations is not computationally feasible. For a complete Agilent 6530 Q-TOF dataset, 180 iterations of seaMass takes 3 h on an Intel Xeon E52630 v3 (2.4Ghz). As expected for sparse decomposition methods, a higher shrinkage parameter converges in less iterations (dotted curves). In particular, the decomposition for a shrinkage of 2^−4^ is similar between 100 and 2000 iterations. For this, the mean absolute error for the final visualisation is around 1.5 ion counts. Note that this is effectively a light denoising of the data, as the decomposition is not capturing the non-smooth, random Poisson noise component. These residuals are only of interest when zoomed in, and can be streamed after the structure has been delivered. The nature of the error is illustrated more closely in Fig. 2B, where the percentage error is plotted across the raw ion count range. Here, it is seen that the percentage error is inflated for data points with ion count less than 10, and reduces as peak intensity increases. A shrinkage of 2^−4^ is used in the remainder of this paper. Figure 2C illustrates the streaming rate for viewports from overview (range 1024 *m/z* by 32 min RT) to deep zoom (4 *m/z* by 2 min) when reading from a standard 7200 rpm hard disk. R-tree decoding from disk was approximately linear but not significantly correlated with viewport size, varying from 118 048 basis functions per second for 4 *m/z* by 2 min to 530 214 for 64 *m/z* by 8 min. Image reconstruction for display at a resolution of 800 × 600 pixels took approximately the same time or was faster than decoding, ranging from 324 399 basis functions per second for 256 *m/z* by 16 min to 1 829 750 for the 4 *m/z* by 2 min zoom. These streaming rates are well within the requirement for real-time interactive visualisations.

**Figure 3:**
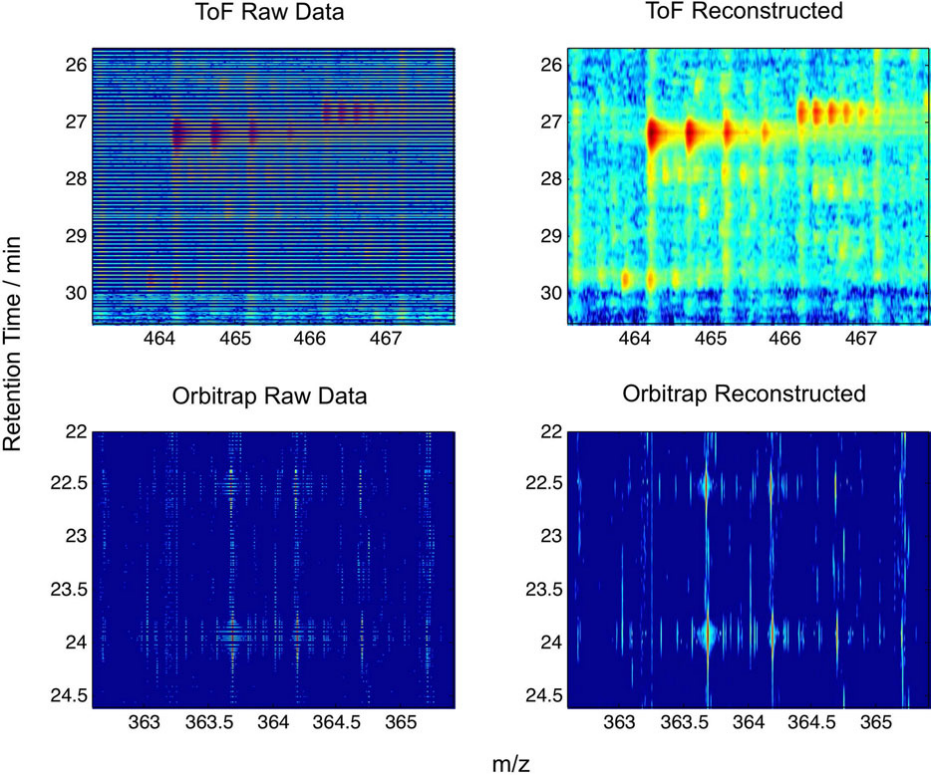
Left: Raw data for a region of a TOF and Orbitrap dataset. DDA MS/MS acquisitions are shown as dark blue (zero counts). Note that Orbitrap data also exhibits expansive regions of zero counts in the MS1 spectra. Right: Surface reconstructions from the generated basis functions, demonstrating the missing data imputation.

Figure 3 shows surface reconstructions of the seaMass signal decomposition from a complete sparse set of basis functions. These images illustrate the effectiveness of the missing data imputation, which relies on seaMass selecting basis functions of maximal scale to fit the available data, which in effect minimises changes in curvature where data is absent.

**Figure 4:**
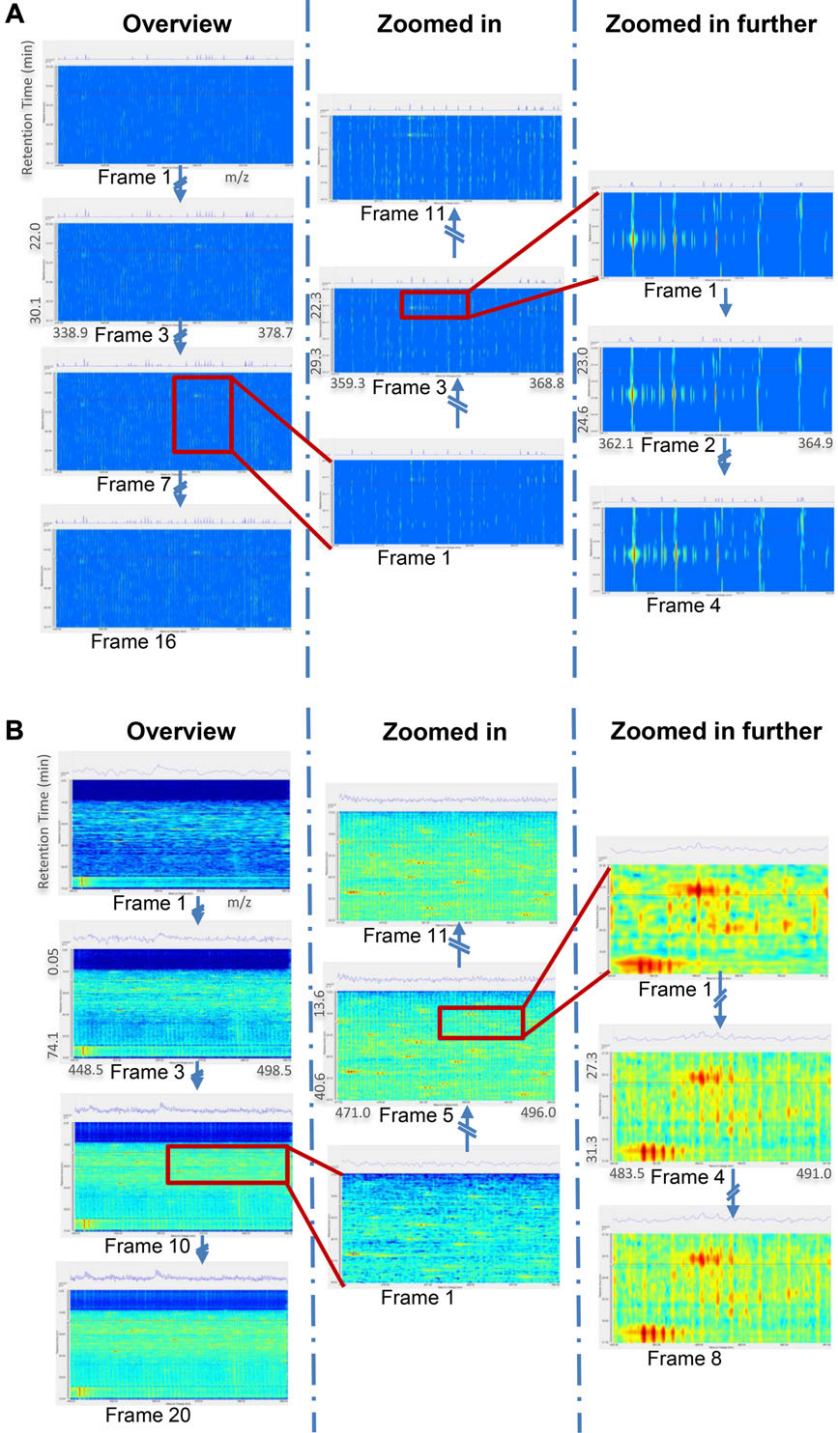
Inspection use case demonstrated on (A) Orbitrap and (B) TOF datasets. In each frame, a 1D view of the spectrum highlighted by a red line in the 2D view is shown, clarifying the incremental data display as basis functions are streamed.

Figure 4 shows how a zooming procedure is handled in our platform. Data visualisation usually starts with an overview of the whole dataset before zooming/panning to specific areas of interest or focusing on specific biochemical signals directly through tables of analysis results. In Fig. 4A, 5000 coefficients are streamed out for each frame of this Orbitrap data. After a few frames, most of the peaks are revealed; only some subtle changes (most of them noise) appear in subsequent frames. It is not necessary to continue panning and/or zooming the dataset until the last frame of the current view displayed: the user can interrupt a data stream at any moment to check other details in a selected region of interest. A similar scenario is observed with the TOF dataset (Fig. 4B), except to note that there is dramatically more background information present in this data. Because of this, 40 000 basis functions were streamed out for each frame update. It can be seen that the overview visualisation does not noticeably improve after ten frames (400 000 basis functions). This is a dramatic reduction on the 14 191 701 data points present in the original.

**Figure 5:**
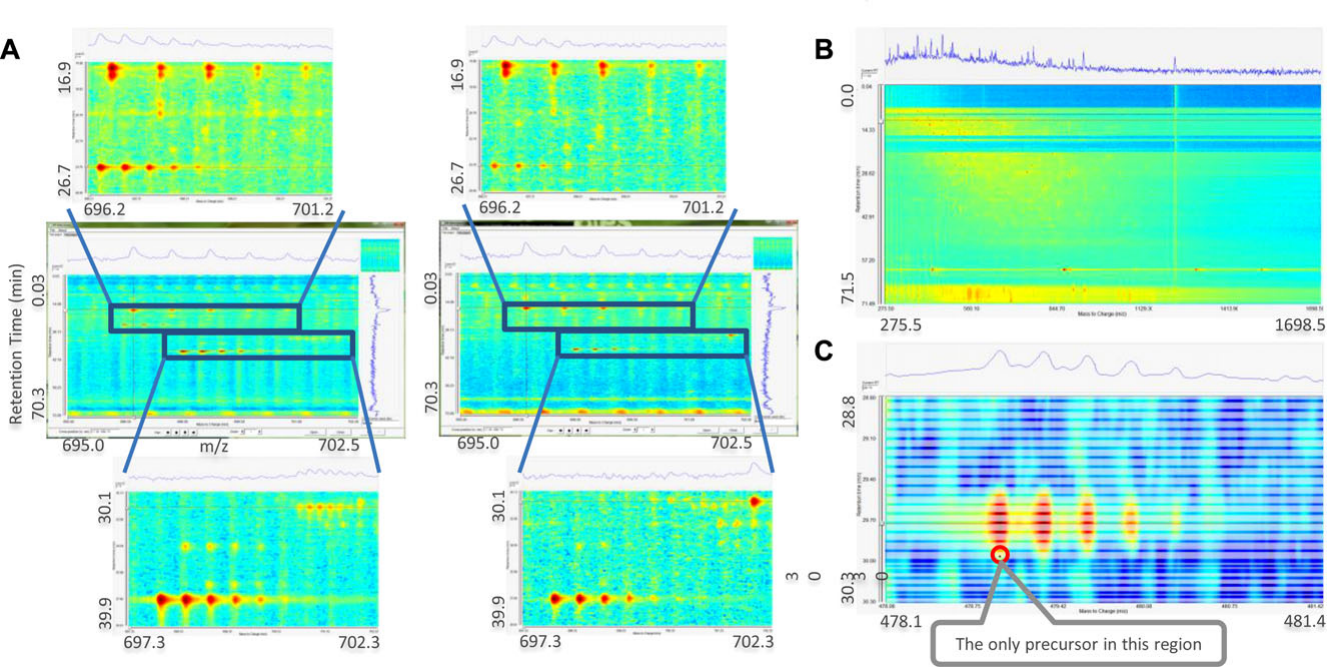
(A) Comparison of two label-free runs to check putative differential expression. (B) This overview display reveals an ionisation issue in the first few minutes of the run. (C) Example of an overlay of MS/MS spectra (translucent areas) and precursor location (black mark) on the imputed reconstruction of a peptide.

Figure 5 demonstrates some potential QC procedures that are facilitated with our platform. Figure 5A compares two small regions of a pair of label-free proteomics runs for differential expression. Coincident peptides are visible that may affect quantification in this region. Figure 5B shows a panorama of a complete run where it can clearly be seen that some problems with the ionisation led to signal dropout in the early stages of the chromatographic run. In Fig. 5C we overlaid the locations of the MS/MS spectra translucently on top of the imputed reconstruction and annotated the precursor location. From this presentation we see that despite the abundance of this visualised peptide, it was almost not selected for fragmentation: only its tail was subject to MS/MS analysis.

## 4 Discussion

This paper presents a novel platform to interactively explore raw profile-mode quantitative LC-MS data with a 2D visualisation similar to Google Maps, but with an asymmetric encoding/decoding scheme specialised to the needs of MS. The LC-MS signal is decomposed into weighted B-spline basis functions that each represent a non-negative baseline component, peak, or peak shoulder. These building blocks are sorted and stored using an R-tree data structure that enables optimised retrieval both spatially and by their overall contribution to the signal.

This computationally intensive but one-time batch pre-processing step is key to subsequent interactive visualisation with minor computational burden (Fig. 2C) and a memory overhead of around 200 Mb (which is predominantly for the R-tree cache). Furthermore, this enables datasets larger than 2 Gb to be opened on computers with 32bit operating systems, which are still very common, whereas existing tools require workstations with large memories in order to load datasets fully, and take minutes to do so. The signal decomposition is also key to the quality of the reconstructions that we can provide with only a small number of the most important basis functions, as illustrated in Figs. 3 and 4. This is crucial to our ensuing goal, which will be to attain remote visualisations across the Internet at interactive and adaptive rates, to demonstrate the potential for seamless access to data stored in public raw data repositories such as ProteomeXchange [1].

In this study, we have shown that our seaMass decomposition approach realises a mean absolute encoding error of around 1.5 ion counts per datapoint. As a sparse signal decomposition approach, seaMass is effectively a transformer coder that is able to achieve statistically lossless compression of LC-MS data. ‘Statistically lossless’ is defined as reflecting the original data within some bound of absolute error. This suggests that our proposed data representation has the merit to potentially become a compressed archival format for LC-MS data similar to the recent NumPress approach [35] but with a significantly more advanced predictive coding scheme and the additional advantage of being visualisation ready. By additionally storing the difference delta between the seaMass reconstruction and the original dataset, full lossless compression would be achieved. Investigation into a joint visualisation and data archival format along these lines is left for future work.

Only 2D visualisation is demonstrated in this paper. 2D visualisation is optimised to show many data points simultaneously. However, it is difficult to grasp intensity variations from just the colour of the points and so 3D visualisation can be useful in providing alternative cues. 3D visualisation support would be entirely a client-side provision and is a topic for future work. Moreover, our signal decomposition algorithm and R-tree data representation provide a solid foundation for 3D visualisation: For a particular viewport, the sorted multiscale weights are mapped to a single scale by using the analytical B-spline operations. Therefore, unlike geometry clipmap or tile-based approaches [17], 3D visualisation based on our data model does not need to handle tile tessellation or crack stitching.

Current proteome informatics and statistical methodology provide detailed and comprehensive reporting, but the scale and opacity of the data can mask unexpected results as well as making it easy to overlook important details. It has recently been proposed that traditional QC procedures can be complemented with new automated tools for generating QC metrics [36]. We suggest that visual analytics integrated into these tools, or provided separately, may aid existing and new QC tasks by harnessing the human advanced visual information processing system [37]. Current LC-MS visualisation tools are limited by their loading and handling of complete datasets, which severely limits productivity and precludes the integrated comparison of whole experiments. In our visualisation engine memory overheads are mitigated, enabling the possibility of implementing novel visualisation schemes integrating results and raw data across complete experiments. This could greatly facilitate QC, verification, validation and expert interpretation of MS analyses, beyond that of what we have illustrated in Fig. 5. If suitable advanced visualisations were developed for MS data analysis, from initial QC to raw data display, peak picking, feature detection, and statistical analysis, analysts may be able to discover not only expected but also unexpected results earlier. The ultimate goal of our platform is therefore to facilitate visual analytics as well as online interactive visualisations for ever-bigger MS datasets.

## Abbreviations

DDA: data-dependent acquisition
LOD: level-of-detail
RT: retention time

## References

[1] Editorial (2012). A home for raw proteomics data. Nat. Meth.

[2] Tao Y, Liu Y, Friedman C, Lussier Y (2004). Information visualization techniques in bioinformatics during the postgenomic era. Drug Discov. Today Biosilico.

[3] Gehlenborg N, O'Donoghue S, Baliga N, Goesmann A (2010). Visualization of omics data for systems biology. Nat. Methods.

[4] Dowsey AW, English JA, Lisacek F, Morris JS (2010). Image analysis tools and emerging algorithms for expression proteomics. Proteomics.

[5] Wang R, Fabregat A, Rios D, Ovelleiro D (2012). PRIDE inspector: a tool to visualize and validate MS proteomics data. Nat. Biotechnol.

[6] Muth T, Weilnbock L, Rapp E, Huber CG (2014). DeNovoGUI: an open source graphical user interface for de novo sequencing of tandem mass spectra. J. Proteome Res.

[7] Li X-J, Pedrioli PGA, Eng J, Martin D (2004). A tool to visualize and evaluate data obtained by liquid chromatography/electrospray ionization/mass spectrometry. Anal. Chem.

[8] Palagi PM, Walther D, Quadroni M, Catherinet S (2005). MSight: an image analysis software for liquid chromatography-mass spectrometry. Proteomics.

[9] Livengood P, Maciejewski R, Chen W, Ebert D (2012). OmicsVis: an interactive tool for visually analyzing metabolomics data. BMC Bioinformatics.

[10] Melamud E, Vastag L, Rabinowitz JD (2010). Metabolomic analysis and visualization engine for LC−MS data. Anal. Chem.

[11] Giannopoulou E, Lepouras G, Manolakos E (2011). Visualizing meta-features in proteomic maps. BMC Bioinformatics.

[12] Martens L, Chambers M, Sturm M, Kessner D (2011). mzML—a community standard for mass spectrometry data. Mol. Cell. Proteomics.

[13] Sturm M, Kohlbacher O (2009). TOPPView: an open-source viewer for mass spectrometry data. J. Proteome Res.

[14] Linsen L, Locherbach J, Berth M, Becher D, Bernhardt J (2006). Visual analysis of gel-free proteome data. IEEE Trans. Vis. Comp. Graph.

[15] Pajarola R, Gobbetti E (2007). Survey of semi-regular multiresolution models for interactive terrain rendering. Visual Comput.

[16] Losasso F, Hoppe H (2004). Geometry clipmaps: Terrain rendering using nested regular grids. Proceedings of ACM SIGGRAPH.

[17] de Corral J, Pfister H (2005). Hardware-accelerated 3D visualization of mass spectrometry data. Proceedings of the IEEE Conference on Visualization.

[18] Chambers MC, Maclean B, Burke R, Amodei D (2012). A cross-platform toolkit for mass spectrometry and proteomics. Nat. Biotechnol.

[19] Starck JL, Murtagh F, Fadili JM (2010). Sparse Image and Signal Processing: Wavelets, Curvelets, Morphological Diversity.

[20] Morris JS, Coombes KR, Koomen J, Baggerly KA, Kobayashi R (2005). Feature extraction and quantification for mass spectrometry in biomedical applications using the mean spectrum. Bioinformatics.

[21] Renard BY, Kirchner M, Steen H, Steen JA, Hamprecht FA (2008). NITPICK: peak identification for mass spectrometry data. BMC Bioinformatics.

[22] Du P, Stolovitzky G, Horvatovich P, Bischoff R (2008). A noise model for mass spectrometry based proteomics. Bioinformatics.

[23] Wang Y, Zhou X, Wang H, Li K (2008). Reversible jump MCMC approach for peak identification for stroke SELDI mass spectrometry using mixture model. Bioinformatics.

[24] Schoenberg IJ (1946). Contributions to the problem of approximation of equidistant data by analytic functions, Part A: on the problem of smoothing of graduation, a first class of analytic approximation. Quart. Appl. Math.

[25] Liao H, Moschidis E, Riba-Garcia I, Zhang Y (2014).

[26] Meagher D (1982). Geometric modeling using octree encoding. Comp. Graph. Image Processing.

[27] Beckmann N, Kriegel HP, Schneider R, Seeger B (1990). The R-star-tree—an efficient and robust access method for points and rectangles. ACM SIGMOD Record.

[28] Saona-Vázquez C, Navazo I, Brunet P (1999). The visibility octree: a data structure for 3D navigation. Comp. Graphics.

[29] Cignoni P, Montani C, Rocchini C, Scopigno R (2003). External memory management and simplification of huge meshes. IEEE Trans Vis. Comp. Graph.

[30] Laine S, Tero K (2011). Efficient sparse voxel octrees. IEEE Trans. Vis. Comp. Graph.

[31] Knoll AM, Wald I, Hansen CD (2009). Coherent multiresolution isosurface ray tracing. Vis. Comp.

[32] Rusu RB, Cousins S (2011).

[33] Elseberg J, Borrmann D, Nüchter A (2013). One billion points in the cloud—an octree for efficient processing of 3D laser scans. ISPRS J. Photogrammetry Remote Sensing.

[34] Unser M, Aldroubi A, Eden M (1993). The L2-polynomial spline pyramid. IEEE Trans. Pattern Anal. Machine Intell.

[35] Teleman J, Dowsey AW, Gonzalez-Galarza FF, Perkins S (2014). Numerical compression schemes for proteomics mass spectrometry data. Mol. Cell. Proteomics.

[36] Tabb DL (2013). Quality assessment for clinical proteomics. Clin. Biochem.

[37] Mukosaka S, Teramoto K, Koike H (2012). mzRepeat: Visual analysis of lipids in mass spectrometry. Proceedings of the IEEE Symposium on Biological Data Visualization.

